# Caregiver involvement in psychiatric inpatient treatment – a representative survey among triads of patients, caregivers and hospital psychiatrists

**DOI:** 10.1017/S2045796020000426

**Published:** 2020-05-22

**Authors:** F. Schuster, F. Holzhüter, S. Heres, J. Hamann

**Affiliations:** 1 Klinikum rechts der Isar der Technischen Universität München, Klinik und Poliklinik für Psychiatrie und Psychotherapie, Munich, Germany; 2 kbo-Isar-Amper-Klinikum München-Ost, Klinik Nord, Munich, Germany

**Keywords:** Families, inpatient psychiatry, quality of care, social support

## Abstract

**Aims:**

Studies on the frequency of caregiver involvement in representative inpatient samples are scarce. The aim of our study was to conduct a representative survey on caregiver involvement in routine inpatient care involving all three parties (patients, caregivers, psychiatrists). Therefore, we performed face-to-face interviews consisting of open-ended questions to gain a deeper understanding of when and how caregivers are involved in care treatment and to identify which topics are mainly discussed.

**Methods:**

This cross-sectional survey included inpatients from 55 acute psychiatric wards across ten psychiatric hospitals, their treating psychiatrists and, when possible, their caregivers. In total, we performed semi-structured face-to-face interviews with 247 patients, their treating psychiatrists and 94 informal caregivers. Each psychiatrist named the next two to three patients to be discharged. After a patient had given informed consent, the interview was performed by a researcher. In addition, the psychiatrist and, when possible, the primary caregiver identified by the patient, were also interviewed.

**Results:**

It was perceived by both patients and psychiatrists that contact between caregiver and psychiatrist had taken place in one-third of the patient cases. Predictors for psychiatrist-caregiver-contact were revealed in the patient's diagnosis (schizophrenia), a lower history of inpatient stays, and the respective hospital. According to psychiatrists the most frequent subjects of discussion with caregivers involved therapeutic issues and organisational and social-psychiatric topics (e.g. work, living and social support). Patients and caregivers stated that psychiatric treatment and the diagnostic classification of the mental illness were the most frequent topics of conversation. For all three groups, the most often cited reason for missed caregiver involvement was the subjective perception that a caregiver was not in fact needed.

**Conclusions:**

Whether or not caregivers were contacted and involved during an inpatient stay strongly depended on the individual hospital. The frequency of involvement of caregivers can certainly be increased by changing processes and structures in hospitals. All three parties (patients, caregivers and psychiatrists) most often stated that the caregiver was not involved in the treatment because they thought it was unnecessary. Evidence demonstrates the positive effect of caregivers' involvement on the therapeutic process but also on the well-being of the caregiver, therefore it is necessary to increase awareness of this evidence among all three interest groups.

## Introduction

Many patients with severe mental illnesses have difficulties with social participation (e.g. working in the primary labour market) and they tend to have less social integration compared to healthy people (Gayer-Anderson and Morgan, 2013; Sundermann *et al*., 2014). Such tendencies of social isolation are one of the reasons why many patients are close to their family and friends and receive support from them in various areas. In this context, family and non-family (e.g. friends and neighbours) caregivers are often referred to as ‘informal carers’ (also referred to as ‘caregivers’) and play a decisive role in the recovery process. In this occasion, caregivers do not only serve as contact persons but provide extensive support in terms of finance and housing (Lester *et al*., 2011; Lavis *et al*., 2015). Furthermore, caregivers assume responsibility for issues that are often insufficiently covered by healthcare professionals (e.g. monitoring medication, improving compliance, etc.) (Jungbauer *et al*., 2004; Lowyck *et al*., 2004). Consequently, informal carers tend to support the affected patient without receiving any financial benefits, thus unburdening the health system and society from paying significant sums of money (Commission, 2012). However, caring for a mentally ill family member unavoidably leads to profound emotional, financial and health burdens for the caregiver (Roick *et al*., 2007; Smith *et al*., 2014). Challenges for caregivers not only arise by long-lasting recovery processes but also by recurrent setbacks characterising many mental illnesses.

There is good evidence that caregiver involvement can have a positive influence on the patients' course of the illness, while at the same time improving the caregivers' own health and well-being (Burns and Kendrick, 1997; Pitschel-Walz *et al*., 2001; Ramirez Garcia *et al*., 2006; Tambuyzer and Van Audenhove, 2013; Dixon *et al*., 2014). In addition, caregiver involvement might be an important factor to facilitate the efficient implementation of shared decision-making processes (Hamann and Heres, 2019). In fact, current studies outline that many caregivers want to be involved in the treatment planning processes. However, they are often uninformed or unaware of how these processes work (Kartalova-O'Doherty and Tedstone Doherty, 2009; Tambuyzer and Van Audenhove, 2013). Joint decision making by patients, caregivers and psychiatrists is not a rule, and complaints are often made regarding ineffective communication and problems with medical confidentiality (Doody *et al*., 2017). Thus, many studies in recent years have concluded that caregivers do not feel sufficiently involved in hospital treatment (Hodgson *et al*., 2002; Wilkinson and McAndrew, 2008).

There is a paucity of research on the frequency of caregiver involvement in representative psychiatric inpatient samples. The few existing studies tend to focus on specific diagnoses (e.g. eating disorders) that are performed without having all three parties involved (patients, caregivers, psychiatrists) in the respective processes, rely solely on questionnaires or are not performed face-to-face (Jubb and Shanley, 2002; Cleary *et al*., 2005; Semrau *et al*., 2016; Reyes-Rodriguez *et al*., 2019).

The aim of our study was therefore to conduct a representative survey on caregiver involvement in routine inpatient care (open and closed wards, day clinics) involving all three parties (patients, caregivers, psychiatrists). Therefore, we performed face-to-face interviews consisting of open-ended questions to gain a deeper understanding of when and how caregivers are involved in care treatment and identify which topics are mainly discussed.

## Method

The present study is a cross-sectional survey of the care provided by 55 acute psychiatric wards in ten psychiatric hospitals in upper Bavaria (Germany) and focuses on the interaction between inpatients, their psychiatrists and when possible, their caregivers.

### Inclusion and exclusion criteria, recruitment of participants

The aim of the present study was to describe patterns of caregiver inclusion for a representative sample of psychiatric inpatients in upper Bavaria. Therefore, participant selection took place on all acute psychiatric state hospital wards (and one psychiatric university hospital) serving the greater Munich area and the southern part of upper Bavaria, covering a catchment area of approximately 4 million inhabitants. Data were collected in the period from October 2018 until August 2019. Wards focusing on elderly psychiatry (65 + years) or alcohol/drug-dependency were not included in the survey. Each psychiatrist was asked for their next two to three patients pending discharge whom were then invited to be interviewed. Invitation was conducted this way in order to cover information regarding caregiver involvement over the entire inpatient stay. In addition, the treating psychiatrist and, when possible, the primary caregiver of the patient were interviewed. The primary caregiver was determined by the patient. In fact, this person was not necessarily a relative but could be of any relation to the patient (informal carer). Semi-structured interviews and standardised questionnaires (outlined further below) were used to gather data on the participants. Each interview was performed once. The only inclusion criterion was that patients treated on a participating ward had to provide a written informed consent. The patient's affirmation provided consent for their own participation as well as for the psychiatrist's participation. Following patients' agreement to participate in this study, an interview was conducted, followed by an interview with the psychiatrist. All patients were additionally asked for their consent to interview their primary caregiver. In cases where patients additionally agreed that a caregiver was contacted, we obtained an informed written consent from the caregiver in order to perform the respective interview. If patients did not consent to contacting their caregiver (but consented to be interviewed themselves), we asked about the underlying reasons for refusal (to contact caregivers) in these cases.

### Data acquisition

We developed structured questionnaires to obtain basic data from the surveys on the three parties (patient, caregiver and psychiatrist). Furthermore, we collected personal data from patients and caregivers (age, sex, marital status, educational background, etc.). In addition, all patients were asked about the existence of a primary caregiver and for their informed consent to contact this person.

Psychiatrists reported patients' clinical data including their diagnosis, duration of illness, number of inpatient stays, etc. and subsequently rated their illness severity (Clinical Global Impression Scale) (Busner and Targum, 2007) and their functional level using the Global Assessment of Functioning (GAF) Scale (Jones *et al*., 1995). Caregivers also provided their personal data (e.g. age, sex, marital status, educational background and family relationship).

Subsequently, we used a structured interview with open-ended questions that covered a spectrum of different aspects pertaining to the caregivers' involvement. In particular, we inquired the level of contact between the treating psychiatrist and the caregiver and how the caregiver was involved in the overall treatment process. Moreover, we asked the three parties (patients, carers and psychiatrists) about the prevalent topics of discussion during psychiatrist-caregiver contact.

### Ethics

The trial was approved by the local review board (Ethikkommission der Technischen Universitaet Muenchen).

### Statistics

Statistical evaluation was performed with IBM SPSS Statistics 25 and it primarily involved the use of descriptive statistics (frequencies, mean values). All subjects included were asked to provide answers to open, semi-open and closed questions. Based on the answers to the open questions, we categorised the respective data for quantitative evaluation. For example, the open-ended responses regarding the content of discussion during psychiatrist-caregiver contact were categorised into: (1) don't know, (2) organisational, social-psychiatric, (3) outpatient psychiatric care, (4) therapy, (5) psychoeducation and (6) diagnostic classification. Additionally, for each open question 30 answers were independently evaluated by two judges. Cohen's kappa coefficients (*κ*) ranged from 0.89 to 0.91, thus indicating an excellent inter-rater reliability (Fleiss, 1981).

To predict caregiver involvement, we performed two logistic regression analyses with distinct dependent variables. The first dependent variable was defined as ‘according to the psychiatrist, there was at least one contact between the caregiver and the psychiatrist during inpatient treatment’ and the second dependent variable as ‘according to the patient, there was at least one contact between the caregiver and the psychiatrist during inpatient treatment’. By using this approach, we were able to investigate psychiatrists' and patients' perspectives with two different logistic regression models separately. Based on expert opinion and a literature review (Cleary *et al*., 2005; Eassom *et al*., 2014; Soklaridis *et al*., 2019), we identified independent variables that were likely to be connected to caregiver involvement. The independent variables for our regression models consisted of sociodemographic patient data (age, sex, mother tongue, etc.), disease-related patient data (diagnosis, GAF, number of inpatient psychiatric stays, etc.) and the treating hospital.

## Results

### Patient sample characteristics

A total of 265 patients were approached to participate in the study, of which most (*n* = 247, 93.2%) agreed. These 247 participants were between 17 and 84 years old, and there were slightly more female than male patients. Roughly half of the patients were treated in a hospital that was classified as urban, whereas the other half was treated in a hospital in a rural region. Approximately two-thirds of the study participants were treated in open wards. About 60% of the patients were diagnosed with an affective (mood) disorder, followed by approximately 30% of patients who were treated with schizophrenia or delusional disorder. The remaining patients were treated for various psychiatric disorders (see Table 1). In all 247 cases, we were able to interview the treating psychiatrist (*n* = 106), and the leading psychologist in some cases.

**Table 1.** Characteristics of patient sample (*n* = 247)

Variable		Frequency (*n*, %)	Dispersion (range, mean, s.d.)
Age			17–84, M = 43.9, s.d. = 15.6
Sex	female	139, 56.3%	
	male	108, 43.7%	
Main Diagnosis	Affective disorder	147, 59.5%	
	schizophrenia or delusional disorder	70, 28.3%	
	Neurotic disorder	11, 4.5%	
	Personality and behavioural disorder	10, 4.0%	
	Psychic and behavioural disorder through psychotropic substances	7, 2.8%	
	Organic psychic disorder	1, 0.4%	
	Huntington's disease	1, 0.4%	
CGI^1^			M = 4.5, s.d. = 1.1
GAF^2^			M = 50.6, s.d. = 15.3
Interview took place on closed/open ward		90, 36.4%/157, 63.6%	
Interview took place in an urban/rural hospital		127, 51.4%/120, 48.6%	

1 Clinical Global Impression

2 Global Assessment of Functioning

### Caregiver recruitment and caregiver sample characteristics

A total number of *N* = 162 participating patients (65.6%) allowed us to contact a caregiver by telephone. The remaining study participants indicated they no longer had an important caregiver (18 patients, 7.3%) or refused to disclose any contact information (67 patients, 27.1%). The most frequent reason for not disclosing contact information was that patients assessed their caregivers to be mentally or physically ill or overburdened by the current situation (*n* = 30). Finally, 18 patients suspected that their caregiver would not participate in this study, and hence refused us permission to contact their caregiver.

For those 162 study participants who named a caregiver and also agreed on their being interviewed, it was possible to establish contact with caregivers in 135 (83.3%) cases. For the remaining 27 patients, the caregiver could not be reached by telephone despite repeated attempts made. Of the 135 persons we were able to contact, 13 (9.6%) refused to participate in our study. Overall only 94 caregivers could be interviewed, as 28 caregivers either did not return their written consent to participate in the study or could no longer be reached for the telephone interview. Conclusively, we were able to interview caregivers in 38.1% of all patient cases.

The 94 caregivers included in this study were aged between 21 and 90 (M = 53, s.d. = 15, 4) and 66.0% were female. Thirty-two caregivers were parents (34.0%), followed by 23 partners (24.5%), 14 siblings (14.9%), six children (6.4%) and three aunts (3.2%). Only 13 important caregivers (13.8%) were unrelated to the patient and three were categorised in the section ‘others’ (3.2%). Finally, only two caregivers stated that they could offer no information about the inpatient treatment of the patient.

### Frequency and predictors of caregiver involvement

The psychiatrists stated that for 83 of the 247 patients (33.6%) they had contact with a primary caregiver at least once. From the patient's perspective *and* the caregiver's perspective, contact between psychiatrists and caregivers had only taken place in 74 (30.0%) and 43 (45.7%) patient cases, respectively (data of the 94 caregivers surveyed). Psychiatrists reported that in 23 cases (27.7%) they had contact with a caregiver without the patient's involvement. Caregivers and psychiatrists were talking face-to-face in 65 patients (78.3%), whereas in 18 cases (21.7%) the involvement was limited to telephone or post.

**Fig. 1.**
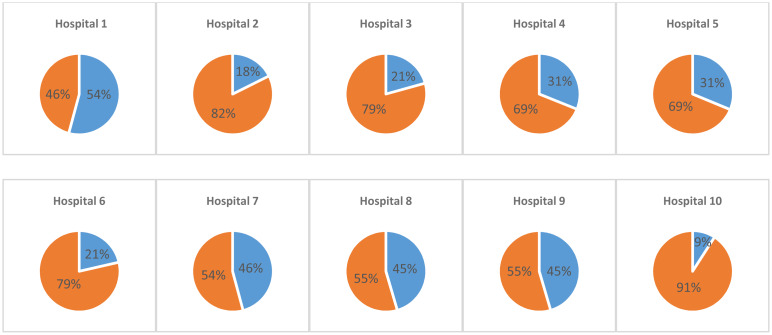
Frequency of caregiver involvement across the ten different hospitals (blue: caregiver involvement, orange: no caregiver involvement).

Contact between caregiver and psychiatrist (psychiatrist's perspective) was predicted by the patient's diagnosis, the number of psychiatric inpatient stays, and the hospital in which the patient was treated (Table 2). As seen in Table 2, caregivers were included more frequently in patients with schizophrenia or delusional disorder. Additionally, the more frequently a patient had been treated in hospital, the less often a caregiver was included in the treatment. Overall, there were substantial differences in caregiver involvement between hospitals (Fig. 1). Contact between caregivers and psychiatrists (patient's perspective) was predicted by the hospital, the diagnosis and the patient's sex (more caregiver involvement for female patients). In both models (patient's and psychiatrist's perspectives) caregiver involvement could be predicted by the hospital and the diagnosis. Alternatively, a link between sex and caregiver involvement could only be seen in the patient's perspective, and a link between the number of previous inpatient stays and caregiver involvement could only be seen in the psychiatrist's perception.

**Table 2.** Predictors for caregiver involvement (logistic regression analyses, multivariable models)

	According to the psychiatrist, there was at least one contact between the caregiver and the psychiatrist during inpatient treatment (*n* = 247)			According to the patient, there was at least one contact between the caregiver and the psychiatrist during inpatient treatment (*n* = 247)		
Variable	*p*-value	OR	95% CI	*p*-value	OR	95% CI
Age	0.91	1.00	0.98–1.02	0.44	1.01	0.99–1.03
Sex (female *v*. male)	0.14	1.61	0.86–2.99	0.03	2.16	1.10–4.26
Native language (German *v*. foreign language)	0.20	0.61	0.28–1.31	0.74	1.15	0.51–2.57
Marital status (married, in partnership *v*. unattached)	0.14	1.67	0.84–3.33	0.14	1.73	0.84–3.55
Mental disorder						
Affective disorder (reference)						
Schizophrenia or delusional disorder	0.00	4.93	2.08–11.67	0.00	3.95	1.56–10.02
Others	0.31	1.65	0.63–4.33	0.51	0.68	0.22–2.13
Global Assessment of Functioning (GAF)	0.59	0.99	0.97–1.02	0.71	1.00	0.97–1.02
Treatment (open ward *v*. closed ward)	0.44	1.36	0.62–3.01	0.28	1.60	0.68–3.75
Number of inpatient psychiatric stays	0.05	0.94	0.87–1.00	0.06	0.93	0.86–1.00
Legal guardian (yes *v*. no)	0.71	0.84	0.34–2.12	0.69	0.82	0.30–2.20
Hospital 1 (reference)						
Hospital 2	0.03	0.18	0.04–0.84	0.02	0.19	0.05–0.80
Hospital 3	0.06	0.28	0.08–1.03	0.00	0.05	0.01–0.23
Hospital 4	0.17	0.49	0.17–1.35	0.00	0.12	0.04–0.36
Hospital 5	0.20	0.46	0.14–1.50	0.01	0.16	0.43–0.57
Hospital 6	0.18	0.33	0.07–1.66	0.03	0.17	0.03–0.87
Hospital 7	0.97	0.98	0.29–3.31	0.12	0.36	0.10–1.28
Hospital 8	0.84	0.85	0.19–3.93	0.04	0.17	0.03–0.94
Hospital 9	0.59	0.65	0.13–3.13	0.21	0.35	0.07–1.82
Hospital 10	0.05	0.10	0.10–0.97	0.01	0.05	0.01–0.49

### Topics of discussion during caregiver involvement

**Fig. 2.**
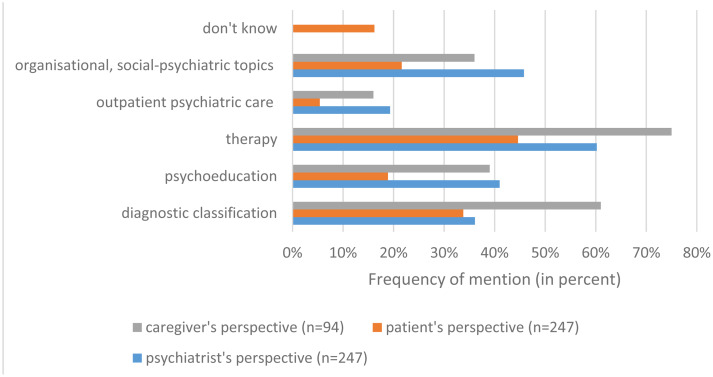
Topics of discussion during caregiver involvement.

Participants' statements as to what had been discussed during caregiver involvement (patient's, caregiver's and psychiatrist's perspective) were categorised in five major topics (see also Fig. 2). From the psychiatrist's perspective, patient's treatment was discussed in 50 cases (60.2%) as opposed to organisational and social issues discussed in 38 cases (45.8%). In all, 34 cases (41.0%) involved discussions involving the caregiver's education regarding the patient's symptoms or illness (‘psychoeducation’), whereas 30 cases (36.1%) involved the caregiver's active participation and support throughout the diagnostic evaluation. In 16 cases (19.3%) outpatient psychiatric care was the content of carer involvement. As illustrated in Fig. 2, the three groups' (patients, caregivers, psychiatrists) perspectives appeared to differ substantially from one another.

### Reasons for no caregiver involvement

**Fig. 3.**
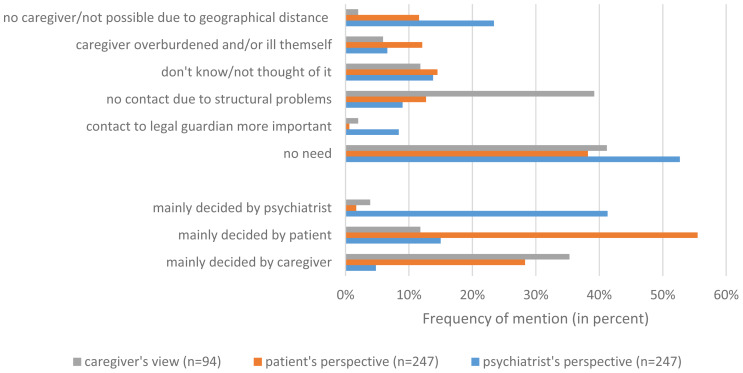
Reasons for no caregiver involvement.

According to the psychiatrist, the two most frequent reasons for not involving a caregiver were the perception that it was unnecessary to involve caregivers in patient care (88 patients, 52.7%) and the unavailability of caregivers owing to geographical barriers (39 patients, 23.4%). According to patients, the most frequent reason for no caregiver involvement was that caregiver involvement was in fact unnecessary (66 patients, 38.2%). Finally, reasons identified by caregivers for their missed involvement were lack of necessity (21 cases, 41.2%) and structural problems (20 cases, 39.2%). Under structural problems we summarised: work overload of the psychiatrics, poor availability, and missing contact information. The majority of patients (*n* = 96, 55.5%) and psychiatrists (*n* = 69, 41.3%) indicated that the decision not to involve a caregiver in treatment was mainly taken by themselves. Caregivers also stated that the decision not to be involved was taken by themselves in 18 cases (35.3%). Figure 3 provides an overview of the reasons that facilitated missed caregiver involvement during patient treatment.

## Discussion

Patients and psychiatrists consistently reported that contact between the primary caregiver and the psychiatrist in charge took place in only one-third of the cases. The most important predictors for psychiatrist-caregiver-contact (patient's and psychiatrist's perspectives) included patients' diagnoses (schizophrenia) and the treating hospital. This is encouraging as family involvement is proven to be effective in the treatment of schizophrenia and treatment guidelines clearly recommend it (Pitschel-Walz *et al*., 2001; Dgppn, 2019). Whereas females were associated with significantly more caregiver involvement in the patient's perspective, this finding did not reach significance in the psychiatrist's perspective. Alternatively, more previous inpatient stays lead to less caregiver involvement in the psychiatric's perspective, this result could not be confirmed from the patient's perspective. According to psychiatrists, and when it comes to caregiver involvement, the most frequent subjects of discussion involved patient's therapeutic issues (e.g. medication, progress in treatment, hospital discharge) and organisational and social-psychiatric topics. On the other hand, patients and caregivers stated that the psychiatric treatment and the diagnostic classification of the mental illness were the most frequent topics of conversation. For all three groups the most often cited reason for missed caregiver involvement was the subjective perspective that caregiver's involvement was considered to be unnecessary.

### Strengths and limitations

This study is one of the few representative psychiatric inpatient surveys on caregiver involvement, focusing on all three parties involved (patients, caregivers and psychiatrists). A particular strength of our study is that all interviews were conducted personally (face-to-face), and thus can provide a broad database. Furthermore, we used questionnaires alongside open-ended questions to gain a deeper understanding of when and how caregivers are involved in care treatment, and to identify which topics are mainly discussed, in a process that eradicates potential bias on the part of the investigators.

Patients who were still severely ill at the time of the interviews provided challenges to our survey because they had difficulties answering our questions. Frequent changes of psychiatrists, as well as patient transfer from one ward to another, also imposed difficulties for the attending psychiatrist to prepare a precise overview of the patient's complete treatment. Finally, owing to the necessity to acquire written informed consent from patients and caregivers, we were only able to include caregivers from approximately 40% of patients.

As one of our key findings, we identified great variance in the frequency of caregiver involvement when comparing different hospitals. In this study we did not gather information to characterise the distinct hospitals in more detail. However, this finding is important and should be the basis for further investigation in a future study. In our opinion, differences are probably partly due to the fact that each hospital has its own philosophy regarding involvement of caregivers. On the other hand, we suspect that caregivers' involvement may, in some cases, be hindered by staff shortages and heavy workloads.

In the section ‘frequency and predictors of caregiver involvement’ we stated: the more frequently a patient had been treated in hospital, the less often a caregiver was included in the treatment. Although we could clearly see a connection, this finding was not significant since the 95% of the odds ratio does overlap 1. Further research with larger sample sizes is needed to obtain more precise estimates and clarify this finding.

We did not survey any self-harm or suicide attempt variables. We expect these variables might also be potential predictors of caregiver involvement and should be investigated in future studies.

In other studies, caregivers often complained that they are not sufficiently involved in hospital treatment (Doody *et al*., 2017), whereas 42% of the caregivers surveyed in our study perceived no need to be involved in the respective treatment. Therefore, it can be inferred that previous studies may suffer from a distinct selection bias (where caregivers of patients suffering from schizophrenia were overrepresented) (Jungbauer *et al*., 2004; Eassom *et al*., 2014). In contrast, our sample is not focused on patients with specific diagnoses and therefore is more representative for general psychiatric inpatient settings. This results in including many caregivers of patients suffering from affective disorders (and higher social functioning).

### Implication for clinical practice

The most important implications for clinical practice arise, in our view, from the profound differences in hospitals' approaches to caregiver inclusion and from the fact that all three interested parties often perceive no or little need for caregiver involvement.

From our observation that caregivers are involved with varying frequency in the treatment provided by distinct hospitals; it can be deduced that involvement is not mainly associated with patient or caregiver characteristics, likewise the treating hospital appears to have a significant influence. Therefore, it is essential to create the necessary requirements in hospitals in order to improve the involvement of caregivers in future. Furthermore, the fact that many participants in our survey (patients, caregivers, psychiatrists) perceive no need for caregiver involvement is contradictory to the current scientific evidence. In fact, this evidence demonstrates that caregiver involvement improves outcomes and well-being for both patients and caregivers (Burns and Kendrick, 1997; Ramirez Garcia *et al*., 2006; Tambuyzer and Van Audenhove, 2013; Dixon *et al*., 2014). Moreover, the analysis of the content of caregiver involvement in our study exhibited that important issues such as diagnostics, therapeutic processes, psychoeducation and outpatient care were discussed. Therefore, to overcome both hurdles it is imperative to disseminate knowledge regarding the potential benefits and prospects of caregiver involvement more efficiently and to improve the implementation of clinical algorithms regarding caregiver involvement. Kaselionyte *et al*. (2019) showed that it is possible to implement a structured approach for caregiver involvement, yet its implementation in practice was more challenging than they had expected. This is exactly why the authors underline the need to employ better and more effective physician support from senior managers and clinical leaders. Dixon *et al*. (2014) used a manualised protocol with shared decision-making processes and principles to accentuate the benefits of caregiver involvement. Researchers and patients outlined their individual therapeutic goals and jointly decided whether a caregiver should be involved or not. This procedure facilitated more frequent contact between researchers and caregivers, but had almost no effect on the frequency of family involvement between the standard treatment team and caregivers (Dixon *et al*., 2014). Both studies demonstrate that a standardised procedure is an essential precondition for the involvement of relatives, which might initially be difficult to implement without additional and more experienced staff. Combining these findings with the results of our study, we propose (among others) the following elements for clinical use as well as for future studies on caregiver involvement:

- Upon admission, obligatory collection of caregiver data
- Distribution of an information sheet (general procedure, benefits etc.) regarding the merits of caregiver involvement to patients and caregivers
- Implementation of at least two basic contacts (on admission, before discharge) with structured agendas (e.g. medical history by caregiver on admission)
- Implementation of caregiver involvement as basic principle within SDM processes (Hamann and Heres, 2019) (adding caregivers as a third party to the process of shared decision making)
- Implementation of family involvement through psychoeducational groups (Rummel-Kluge *et al*., 2006; Frank *et al*., 2014)
- Provide distinct training to hospital staff to better understand the merits stemming from caregiver involvement and from standardised procedures in this topic

Similar to other approaches (e.g. Safewards in the context of coercive practices in mental health (Bowers, 2014)) the combination of these elements might be necessary to change existing practice patterns.

To implement such a concept in psychiatric inpatient care it would be necessary to announce a person in charge. After an initial training this person could pass on knowledge and establishes workflows one after another. We believe a step by step approach would thereby increase feasibility.

In conclusion it can be said: In the patients' and the psychiatrists' perspectives, caregiver involvement takes place in only about one-third of the cases. Predictors for psychiatrist-caregiver-contact included the patient's diagnosis, the number of previous inpatient stays, the patient's sex and the respective hospital. All three parties (patients, caregivers and psychiatrists) most often stated that the caregiver was not involved in the treatment because they thought it was unnecessary. This finding is in contrast with broad scientific evidence, which suggests benefits in caregiver involvement. In order to substantially increase caregiver involvement, we believe it is essential to increase awareness of this evidence in all three interested parties.

## Data

Data will be available upon reasonable request.
